# Endotoxin and cytokines in patients with gastrointestinal tract perforation

**DOI:** 10.1155/S0962935192000097

**Published:** 1992

**Authors:** S. Endo, K. Inada, Y. Inoue, T. Otsu, T. Kasai, Y. Kuwata, S. Hoshi, M. Yoshida

**Affiliations:** 1 Critical Care and Emergency Center School of Medicine Iwate Medical University 19-1 Uchimaru Morioka 020 Japan; 2 Department of Bacteriology School of Medicine Iwate Medical University 19-1 Uchimaru Morioka 020 Japan

## Abstract

Plasma levels of endotoxin and various cytokines were assessed in 70 patients with gastrointestinal tract perforation. Sepsis developed in 29 of them, and eight of these (27.6%) had on admission endotoxin levels higher than 9.8 pg ml^-1^. The clinical outcome correlated with the level of tumour necrosis factor α (TNFα), rather than with the endotoxin level. The high interleukin 6 (IL-6) level was shown in septic patients and no correlation was observed between the IL-6 level and the clinical outcome. Plasma TNFα levels tended to change independently from endotoxin levels, suggesting that TNFα may have been locally produced in inflammatory lesions.


Mediators of Inflammation 1, 45–48 (1992)

### Endotoxin and cytokines in patients with gastrointestinal tract perforation

S. Endo,[1,CA] K. Inada,[2] Y. Inoue,[1] T. Otsu,[1] T. Kasai,[1] Y. Kuwata,[1] S. Hoshi[1] and M. Yoshida[2]

[1]Critical Care and Emergency Center, and [2]Department of Bacteriology, School of Medicine, Iwate Medical University, 19-1 Uchimaru, Morioka 020, Japan.

[CA] Corresponding Author

**PLASMA levels of endotoxin and various cytokines were assessed in 70 patients with gastrointestinal tract perforation. Sepsis developed in 29 of them, and eight of these (27.6%) had on admission endotoxin levels higher than 9.8 pg ml$^{-1}$. The clinical outcome correlated with the level of tumour necrosis factor α (TNFα), rather than with the endotoxin level. The high interleukin 6 (IL-6) level was shown in septic patients and no correlation was observed between the IL-6 level and the clinical outcome. Plasma TNFα levels tended to change independently from endotoxin levels, suggesting that TNFα may have been locally produced in inflammatory lesions.**

**Key words:** Endotoxin, Gastrointestinal tract perforation, Interleukin 6, Tumour necrosis factor α

### Introduction

Diffuse peritonitis occurring after perforation of the gastrointestinal tract is associated with serious complications such as sepsis, septic shock, disseminated intravascular coagulation (DIC), adult respiratory disease (ARDS), and multiple organ failure (MOF). Endotoxin derived from the cell walls of gram-negative bacteria has traditionally been believed to be responsible for such complications. However, recent attention has been paid to the various cytokines, which may play a significant role as mediators in the induction of such pathological conditions.[1–3]

We monitored plasma endotoxin and cytokine levels in patients with gastrointestinal tract perforation in whom blood could be collected from the earliest stage of the disease.

### Materials and Methods

*Patients:* This study was performed in a total 70 patients with gastrointestinal perforation (excluding injury and appendicitis) who were admitted to our centre in the 3 year period from October 1987 to September 1990, and in whom blood collection was possible from the earliest stage after perforation. There were 45 cases of gastroduodenal perforation, twelve cases of small bowel perforation, and 13 cases of colonic perforation. Patients without sepsis were classified as group A, those who developed sepsis and survived formed group B, and those who developed sepsis and died were placed in group C.

The diagnosis of sepsis was based on clear detection of focus of infection, positive blood culture, temperature higher than 38.5°C or lower than 35.5°C, or a white blood cell count more than 15 000 or less than 3 000.

*Blood collection:* Blood samples were collected on admission and every day thereafter. When any serious clinical change, including shock, DIC, ARDS, and MOF, arose in the patients, blood was collected about every 2–3 h. Blood was collected into heparinized containers and centrifuged at 3 000 rpm for 40 s. The plasma samples thus obtained (PRP) were stored at −80°C until required for analysis.

*Measurement of endotoxin and cytokines:* Endotoxin levels were measured using the endotoxin-specific Endospecy test (Seikagaku Corporation, Tokyo, Japan).[4] The new PCA method[5,6] that we devised ourselves was used for the pretreatment of plasma samples. The normal range of plasma endotoxin levels is below 9.8 pg m$^{-1}$ with this assay.

Plasma was diluted to a two-fold volume, then TNFα and IL-6 levels were measured with ELISA (Genzyme MA, USA). The detection limits for TNFα and IL-6 were 0.024 ng ml$^{-1}$ and 0.3 ng ml$^{-1}$, respectively.

*Statistical analysis:* Data are expressed as the mean ± SD. Statistical analysis was performed with the two-tailed Wilcoxon rank-sum test for unpaired data, and the level of significance was set at $p < 0.05$.

### Results

The endotoxin level exceeded 9.8 pg ml$^{-1}$ in eleven of the 70 patients with gastrointestinal

© 1992 Rapid Communications of Oxford Ltd

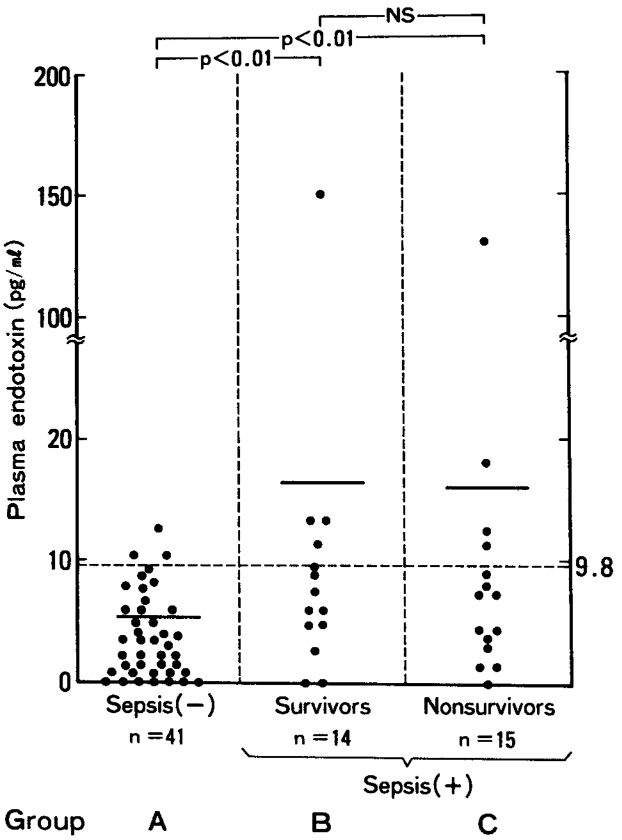

FIG. 1. Plasma endotoxin levels in groups A, B, and C. The normal plasma endotoxin level is below 9.8 pg $ml^{-1}$.

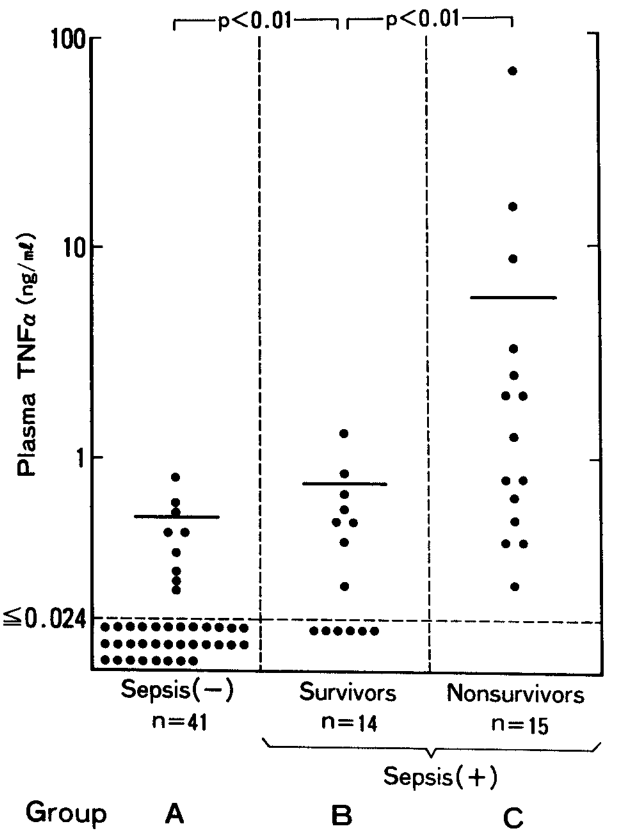

FIG. 2. Plasma TNFα levels in groups A, B, and C. The detection limit of TNFα is 0.024 ng $ml^{-1}$.

perforation (15.7%) (Fig. 1). Sepsis occurred in 29 patients, and eight of them had an endotoxin level higher than normal (27.6%). The eleven patients with abnormally raised endotoxin levels comprised three were in group A (3/41; 7.3%), four in group B (4/14; 28.6%), and four in group C (4/15, 26.7%). The mean endotoxin level was significantly higher in group B and C than in group A.

The septic patients comprised nine cases of gastrointestinal perforation, another nine of small bowel intestinal perforation, and eleven cases of colonic perforation. The septic patients who had an endotoxin level exceeding 9.8 pg $ml^{-1}$ included one of the nine cases of gastroduodenal perforation (11.1%), two of the nine cases of small bowel intestinal perforation (22.2%), and five of the eleven cases of colonic perforation (45.5%). Thus, the incidence of abnormal endotoxin levels was significantly higher in the patients with colonic perforation than in those with perforation at other gastrointestinal sites.

There was no significant difference in the incidence of abnormal endotoxin levels between groups B and C.

The TNFα level exceeded the detection limit in 9/41 patients in group A (22.0%), 8/14 patients in group B (57.1%), and all 15 patients in group C (100%). The mean TNFα level was significantly higher in group B than in group A, and also significantly higher in group C than in group B (Fig. 2).

The IL-6 level exceeded the detection limit in 7/41 patients in group A (17.1%), 12/14 patients in group B (85.7%), and 13/15 patients in group C (86.7%). The mean IL-6 level was significantly higher in groups B and C than in group A, but there was no significant difference between group B and C (Fig. 3).

### Discussion

It has generally been believed that diffuse peritonitis due to gastrointestinal tract perforation is associated with various complications because of the high plasma endotoxin levels present in such patients. The previously available methods for detecting endotoxin were not specific and false-positive readings could be produced by various substances, so that results obtained with them may not have been accurate. The endospecy test[1] is an endotoxin-specific assay that was developed recently. However, a problem still remained with the conventional method for treating plasma. With the existing PCA method for the pretreatment of plasma, only one eighth of the actual endotoxin level was detected, because the precipitate produced

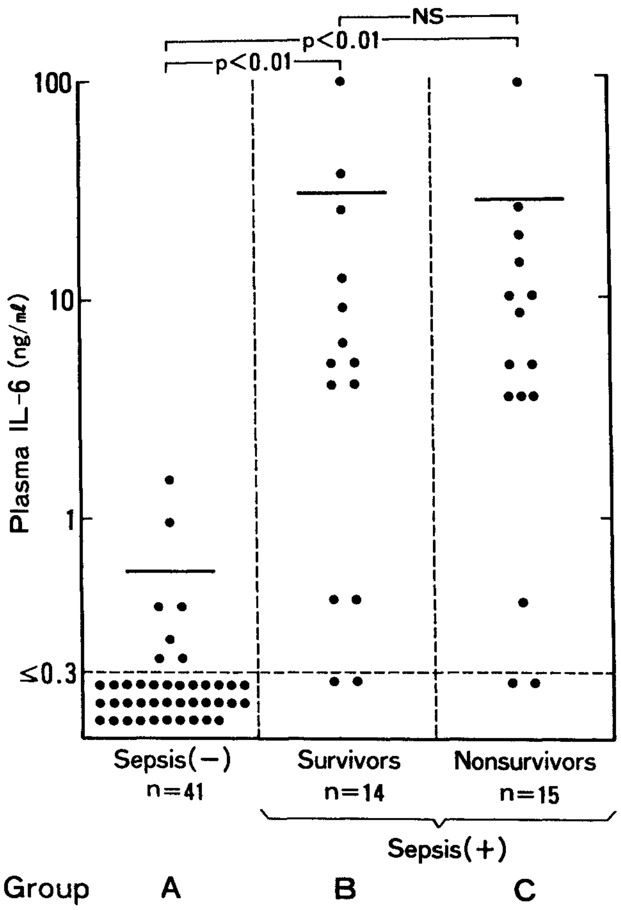

FIG. 3. Plasma IL-6 levels in groups A, B, and C. The detection limit of IL-6 is 0.3 ng $ml^{-1}$.

during treatment and which contained a large amount of endotoxin, was discarded.[5,6] Thus, the relationship between various pathological states and plasma endotoxin levels could not be accurately determined. We therefore devised a new PCA method[5,6] which allowed us to also measure the endotoxin in the precipitate by melting the precipitate obtained from plasma.

The incidence of endotoxemia shown in this investigation was much lower than expected. However, it appears to be appropriate that the incidence of endotoxemia was higher in patients with small bowel perforation than in those with gastroduodenal perforation, and that it was higher again in patients with colonic perforation than in those with small bowel perforation. Comparison between the survivors and those dying of bowel perforation did not show any significant difference in the incidence of high endotoxin levels or in the mean endotoxin level. This finding is inconsistent with the traditional concept that endotoxin is closely related to the promotion of complications after bowel perforation. To explain the low incidence of endotoxemia in patients with diffuse peritonitis arising from gastrointestinal perforation, it could be presumed that bacterial invasion outside the bowel is infrequent or that it produces endotoxin levels below 9.8 pg $ml^{-1}$ in most cases. It may be also possible that the intestinal barrier is so strong that endotoxemia does not occur.

The TNFα level following gastrointestinal perforation was significantly higher in patients with sepsis (groups B and C) than in those free from septic complications (group A). Furthermore, the TNFα level in the septic patients who died (group C) was significantly higher than that in the septic patients who survived (group B). These results suggested that TNFα had a significant involvement with the progression of disease after intestinal perforation.

In the patients of groups B and C, no correlation was shown between the plasma endotoxin and TNFα levels, and the plasma levels of these substances changed independently of each other. The possibility was thus suggested that TNFα in plasma is produced locally at the site of infection due to induction by endotoxin or other stimulatory substances.

Mozes *et al.*[7] have reported that in the experimental endotoxic shock, the TNF level rather than the endotoxin level has an important role in the prognosis.

IL-6 has various biological activities, e.g., it is a major mediator of inflammation and can be detected in various inflammatory responses.[8] Especially in sepsis or septic shock, IL-6 is thought to interact with endotoxin, IL-1, and TNF, and to play a significant role in various pathological changes. These substances enhance the production of each other via feedback loops and form a complex cytokine network.[9] In this study, the IL-6 level was significantly higher in the septic patients (groups B and C) than in the non-septic patients (group A), suggesting the involvement of IL-6 in inflammation. IL-6 levels showed no significant difference between the survivors (group B) and the patients dying of sepsis (group C). Septic shock has been reported in patients with high levels of both IL-6 and IL-1β.[10] The finding in this study suggested that in patients with gastrointestinal perforation, endotoxin and cytokines (which are produced by inflammatory stimulation) both have an extensive involvement in the course of disease due to their complex interactions.

### References

1. Tracey KJ, Beutler B, Lowry SF, *et al.* Shock and tissue injury by recombinant human cachectin. *Science* 1986; **234**: 470–474.
2. Okusawa S, Gelfand JA, Ikejima T, *et al.* Interleukin-1 induces shock-like state in rabbits: synergism with tumour necrosis factors and effect of cyclo-oxygenase. *J Clin Invest* 1988; **81**: 1162–1172.
3. Endo S, Inada K, Inoue Y, *et al.* Interaction between endotoxin and cytokines in septic shock in humans (in Japanese). *J Jpn Assoc Surg Trauma* 1991; **5**: 163–171.
4. Obayashi T. Addition of perchloric acid to blood samples for colorimetric limulus test using chromogenic substrate: comparison with conventional procedures and clinical applications. *J Lad Clin Med* 1984; **104**: 321–330.

5. Takahashi K. Study on quantitative measurement of endotoxin in human blood using chromogenic substrate—especially pretreatment of plasma (in Japanese). *J Iwate Med Ass* 1988; **40:** 67–81.
6. Inada K, Endo S, Takahashi K. *et al.* Establishment of a new perchloric acid treatment method to allow determination of the total endotoxin content in human plasma by the limulus test and clinical application. *Microbial Immunol* 1991; **35:** 303–314.
7. Mozes T, Ben-Efraim S, Tak CJAM, *et al.* Serum levels of tumor necrosis factor determine the fatal or no fatal course of endotoxic shock. *Immunol Lett* 1991; **27:** 157–162.
8. Houssiau FA, Devogelaer JP, Van Damme J, *et al.* Interleukin 6 in synovial fluid and serum of patients with rheumatoid arthritis and other inflammatory arthritis. *Arthritis Rheum* 1988; **31:** 784–788.
9. Shalaby MR, Waage A, Aarden I., *et al.* Endotoxin, tumor necrosis factor-α and interleukin 1 induce interleukin 6 production *in vivo*. *Clin Immunol Immunopathol* 1989; **53:** 488–498.
10. Endo S, Inada K, Inoue Y, *et al.* Septic shock induced by interleukin 1β and interleukin 6 (in Japanese). *JJAAM* 1991; **2:** 733.

ACKNOWLEDGEMENTS. The authors wish to thank Miyuki Suzuki and Naohiko Yamashita for their technical help. This study was supported, in part, by grants from the Ministry of Education, Culture and Science of Japan, and from the Marumo Critical Care Medicine Research Foundation.

**Received 10 October 1991; accepted in revised form 13 December 1991**
